# Supplementary figures and images for: Engagement in water governance action situations in the Lake Champlain Basin
Source: PLoS One. 2023 Mar 16;18(3):e0282797. doi: 10.1371/journal.pone.0282797 (PMC10019647; doi:10.1371/journal.pone.0282797)

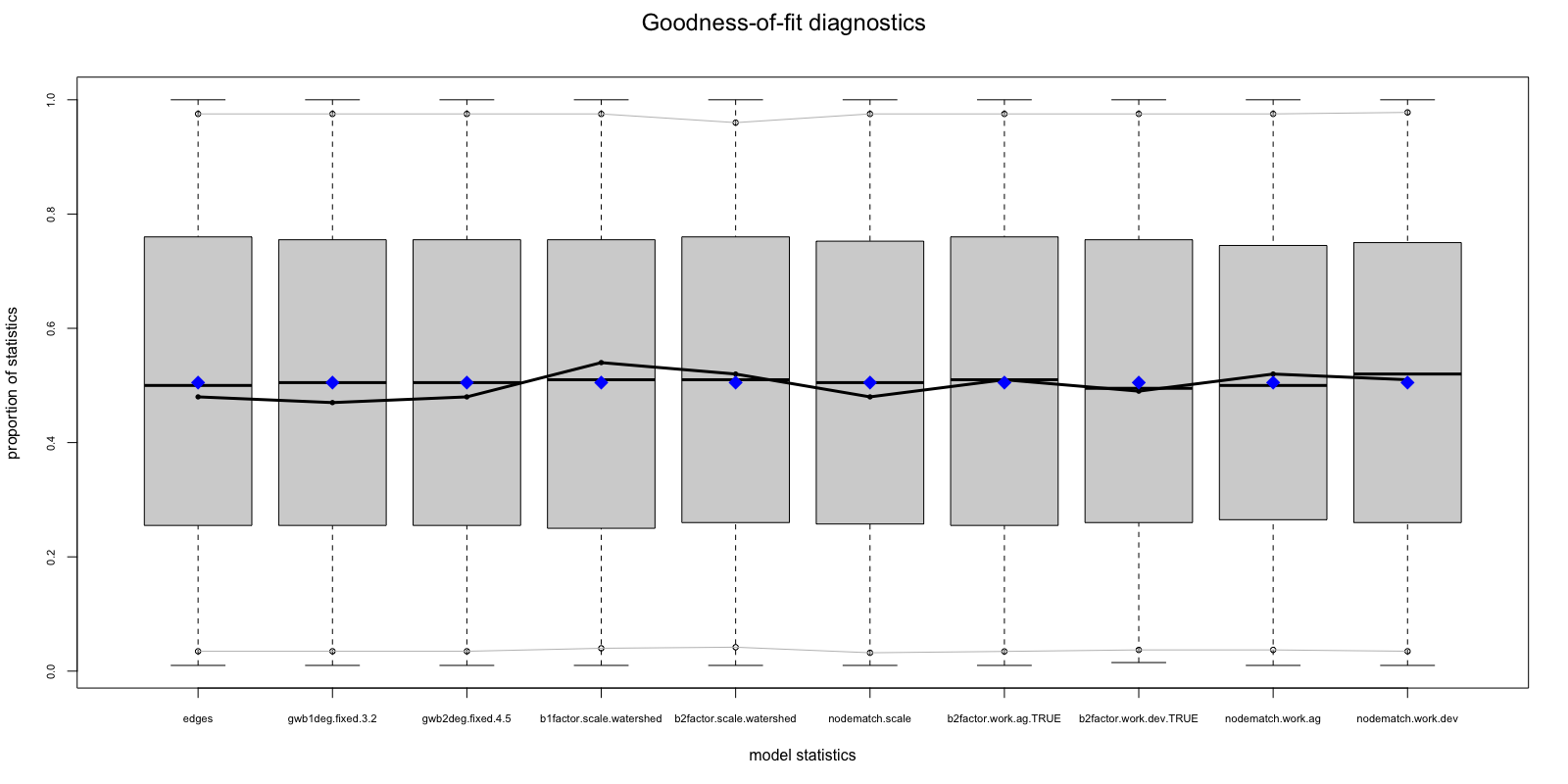

Supplement: S1 Fig — (TIF) [file pone.0282797.s001.tif]

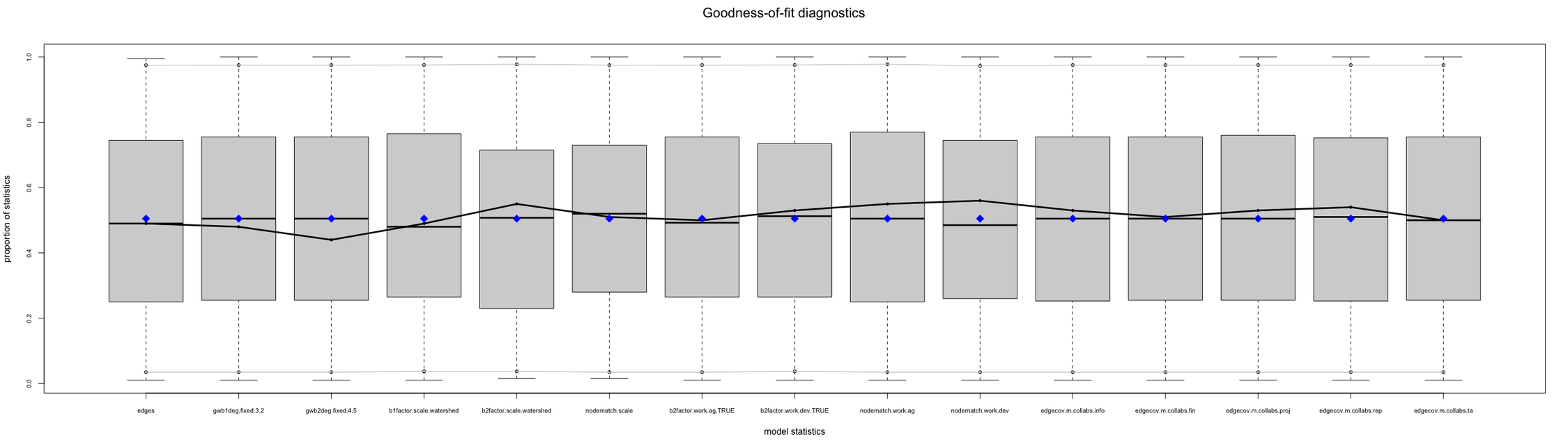

Supplement: S2 Fig — (TIF) [file pone.0282797.s002.tif]
