# Supplementary material for: Engagement in water governance action situations in the Lake Champlain Basin
Source: PLoS One. 2023 Mar 16;18(3):e0282797. doi: 10.1371/journal.pone.0282797 (PMC10019647; doi:10.1371/journal.pone.0282797)
Supplement: S1 File — (PDF) [file pone.0282797.s003.pdf]

Supporting information

"Actor scale" corresponds to the coded scale (i.e., watershed or LCB) assigned to each actor in the network. This parameter is positive and significant for actors at the watershed scale, indicating that watershed-scale actors are more likely to participate in action situations.

"Action situation scale" corresponds to the coded scale (i.e., watershed or LCB) assigned to each action situation in the network. This parameter is positive and significant for actors at the watershed scale, indicating that watershed-scale action situations are more likely to attract participation from actors in the network.

"Action situation factor" corresponds to the coded issues assigned to each action situation in the network. These parameters are negative and significant for agricultural action situations, indicating that agriculture-related action situations are less likely to attract participation from actors in the network.

For $dyad_{i,j}$, the "issue homophily" parameters measure whether $actor_i$ and and action $situation_j$*'s* focus on particular water quality issues affects the likelihood of participation. Agricultural issues show no significant homophily, but we measure positive and significant homophily for development issues.

For $dyad_{i,j}$, the "scale homophily" parameters measure whether $actor_i$ and and action $situation_j$*'s* spatial scale affects the likelihood of participation. In the first ERGM, we find that scale homophily is both positive and significant. In the second, expanded model, this term is not significant, but given the stated issues with model fit, we exclude this model from any conclusions.

The "edges" parameter measures the number of edges in the network. It is equivalent to an intercept term in a logistic regression.

"gwb1degree" is a control parameter equal to the weighted degree distribution of actors in the bipartite graph, subject to a decay parameter. To fit the models (see diagnostic graphs below), we used a decay of 3.2 In both models, the parameter was negative and significant. This supports our conclusion that actors do not tend to participate in the same action situations as those they already collaborate with. We discuss this at length in the discussion section.

"gwb2degree" is a control parameter equal to the weighted degree distribution of action situations in the bipartite graph, subject to a decay parameter. To fit the models (see diagnostic graphs below), we used a decay of 3.2 In both models, the parameter was negative and significant. This supports our conclusion that actors do not tend to participate in the same action situations as those they already collaborate with. We discuss this at length in the discussion section.
The "edgecov" terms correspond to edge covariates for each edge in the network. In this case, for each actor-action situation edge, and for each of the 5 modes of interaction (i.e.,

information sharing, project coordination, technical assistance, reporting, financial exchange) we calculated the number of actors connected to the focal actor that also participate in the same action situation.
